# Adsorption Potential, Speciation Transformation, and Risk Assessment of Hg-, Cd-, and Pb-Contaminated Soils Using Biochar in Combination with Potassium Dihydrogen Phosphate

**DOI:** 10.3390/molecules29102202

**Published:** 2024-05-08

**Authors:** Dun Wu, Jianwei Lu, Kun Huang, Longjin Jiang, Xia Gao, Shuqin Li, Hai Liu, Boren Wu

**Affiliations:** 1 Anhui Provincial Key Laboratory of Intelligent Underground Detection, State Key Laboratory of Safety and Health for Metal Mines, College of Civil Engineering, Anhui Jianzhu University, Hefei 230601, China; lu1245076898@163.com (J.L.); kunhuangah@ahjzu.edu.cn (K.H.); 2 School of Earth and Space Sciences, University of Science and Technology of China, Hefei 230026, China; 3 Anhui ChaoYue Environmental Protection Technology Co., Ltd., Chuzhou 239000, China; jianglongjin@ah-cy.cn; 4 School of Architecture and Urban Planning, Anhui Jianzhu University, Hefei 230601, China; gaoxia@ahjzu.edu.cn; 5 Sinosteel Maanshan General Institute of Mining Research Co., Ltd., Maanshan 243000, China; lishuqin03@foxmail.com; 6 Public Geological Survey Management Center in Anhui Province, Hefei 230091, China; nftslh@gmail.com (H.L.); wuboren66@163.com (B.W.)

**Keywords:** adsorption potential, risk assessment, contaminated soil, biochar, KH_2_PO_4_

## Abstract

The objective of this study is to develop a remediation technology for composited heavy metal-contaminated soil. Biochars (BC300, BC400, and BC500) derived from corn were combined with potassium dihydrogen phosphate (KH_2_PO_4_) to immobilize and remove heavy metal ions, including mercury (Hg^2+^), cadmium (Cd^2+^), and lead (Pb^2+^). The adsorption kinetics of metal ions in aqueous solutions with different concentrations was tested, and the fitting effects of the two models were compared. The findings demonstrate that the joint application of biochar and KH_2_PO_4_ could markedly enhance the immobilization efficacy of Pb^2+^, whereas the utilization of KH_2_PO_4_ on its own exhibited a more pronounced immobilization impact on Cd^2+^. Furthermore, the present study underscores the shortcomings of various remediation techniques that must be taken into account when addressing heavy metal-contaminated soils. It also emphasizes the value of comprehensive remediation techniques that integrate multiple remediation agents. This study offers a novel approach and methodology for addressing the intricate and evolving challenges posed by heavy metal contamination in soil. Its practical value and potential for application are significant.

## 1. Introduction

With the development of economic activities, mining and agricultural processes are releasing large amounts of heavy metals into soils [1,2]. The growing recognition of heavy metal contamination in soil as a critical issue impeding the healthy urbanization process in numerous countries underscores the urgency of addressing this problem. These pollutants are characterized by their non-biodegradability, prolonged persistence, high toxicity, and potent bioaccumulation capabilities. They not only disrupt the structure and functionality of soil ecosystems but also pose a significant threat to human health through their accumulation in the food chain [3]. Therefore, the development of a straightforward, cost-effective, and high-performing remediation technique for heavy metal-contaminated soil is of paramount importance.

Moreover, natural soils often contend with the challenge of combined heavy metal pollution, as well as the complex interactions between various heavy metals and the intricate dynamics at the soil interface [4]. In light of these complexities, the synergistic application of multiple remediation agents may prove to be more efficacious in the passivation of heavy metals, which provides a promising strategy for addressing the multifaceted nature of soil contamination. Dong et al. [5] employed an Fe_2_O_3_–vermiculite (Fe-V) composite material to passivate the co-contaminated soil with As and Cd. In the study group experiments, the toxicity reduction rates of As and Cd were, respectively, higher than 90% and 80–100% in soil A (As: 45 mg/kg and Cd: 6 mg/kg). And in soil B (As: 80 mg/kg and Cd: 10 mg/kg), the toxicity reduction rates of As and Cd were over 84.68% and 99%, respectively. In the study of Zhao et al. [6], superphosphate, humic acid, and fly ash and their complex combination were adopted to passivate the artificially simulated Pb- and Cd-containing soils. Their study revealed that all of the remediation techniques, with the exception of adding humic acid alone, were capable of diminishing the concentrations of Pb and Cd in soils through extraction by CaCl_2_ and diethylenetriaminepentaacetic acid (DTPA). The most effective approach for reducing these concentrations was identified as the sequential application of superphosphate followed by humic acid, with fly ash being the subsequent choice. Lyu et al. [7] found that MnO*_x_* loading can significantly increase the number of functional groups (e.g., hydroxyl, carboxyl, and phenolic hydroxyl groups) on the surface of biochar. The immobilization rate of MnO*_x_*/biochar on Cd, Pb, Zn, and Cu in the soil was two to four times that of unmodified biochar at a MnO*_x_*/biochar dosage of 1%. Fu et al. [8] applied iron-impregnated wheat straw biochar to reconstruct the Cd, Cu, and As co-contaminated soil. The findings of the experiments indicated that the use of passivated materials enhances the conversion of Cd, Cu, and As in soil from an active state to a potentially active or stable state. Furthermore, the remediation efficiency for soils contaminated with heavy metals and As using this modified material is superior to that achieved with unmodified biochar. Incubation experiments from Li et al. [9] showed that phosphorus-passivated biochars enhanced the transformation of Cu (II) and Cd (II) ions from acid-soluble forms to more stable forms, which decreased the extraction of Cu (II) and Cd (II) by two to three times. This was mainly because phosphorus compounds in passivated biochar play a vital role in immobilizing Cu (II) and Cd (II) by forming precipitates or complexes with them.

In recent years, the development and refinement of green mining concepts have led to significant improvements in the environmental quality of areas surrounding mines, particularly in terms of addressing heavy metal pollution. However, more research is needed to understand how to effectively remediate complex heavy metal co-contamination and elucidate the underlying mechanisms. The current literature reveals a lack of studies on composites passivated with biochar and phosphate fertilizers [10,11,12,13]. Despite this, remediation techniques that use blended amendments for heavy metal-contaminated soils have great practical potential due to the complexity and variability of heavy metal pollution in natural soils. Phosphate materials are known to be cost-effective and powerful agents for chemical remediation [8], and biochar, with its excellent adsorption capabilities and abundance of oxygen-containing functional groups, has been widely used in the remediation of soils affected by heavy metals [9,14]. However, the different characteristics of various heavy metals result in different interaction dynamics with biochar. This study proposes the use of the “biochar + KH_2_PO_4_” combined remediation technique for soils in the Tongling mining area which contain high levels of heavy metals such as cadmium (Cd), lead (Pb), and mercury (Hg) due to mining and metallurgical operations. The “biochar + KH_2_PO_4_” method offers certain practical advantages, taking advantage of the synergistic effects between different restorative agents. This approach not only mitigates soil acidification associated with phosphate application but also effectively passivates and remediates pollution [15,16,17]. The integration of these amendments provides a robust and multifaceted strategy for the remediation of heavy metal-contaminated soils, especially in areas with a history of intense mining activities.

In this paper, not only were soil remediation experiments carried out under laboratory conditions, but demonstration studies were also carried out under field conditions to verify the remediation effect of the biochar and KH_2_PO_4_ complex on heavy metal-contaminated soil. The chemical speciation of heavy metals in the soil was analyzed by using the modified Tessier sequential extraction procedure, and the remediation effect was evaluated by employing the toxicity characteristic leaching procedure (TCLP), which provided a more comprehensive assessment method for soil remediation. These innovations demonstrated the potential of a biochar and KH_2_PO_4_ complex in the remediation of heavy metal-contaminated soil and provided a new strategy and theoretical basis for the development of soil remediation technology.

## 2. Results and Discussion

### 2.1. Physicochemical Properties of Corn Straw Biochar

The physicochemical properties of corn straw biochar prepared at three temperatures are listed in Table 1. With the increase in temperature, the pH value of the biochar increased, and the ash content gradually increased. The pH value of the biochar rose as the temperature climbed, as did the ash content. The pH values of the three biochars were 7.49, 8.95, and 9.85, increasing from weakly alkaline to alkaline, respectively, while the ash content of the biochars rose from 21.31 to 24.54%. Previous studies showed that biochar is generally alkaline. In biochar, carbonates, inorganic alkali salts, alkali metal ions in ash [14,15], and deprotonated oxygen-containing functional groups [9,16] are the primary source of alkalinity. EC reflects the content of soluble salts in biochar, which differs from the changing trend of pH value and ash content. BC400 had the highest EC value at 5.74 ms/cm, while BC500 had the lowest at 2.79 ms/cm. The shifting pattern in water-soluble phosphorus content and EC was comparable, indicating that water-soluble phosphorus might be one of the critical sources of EC in biochar.

**Figure 1:**
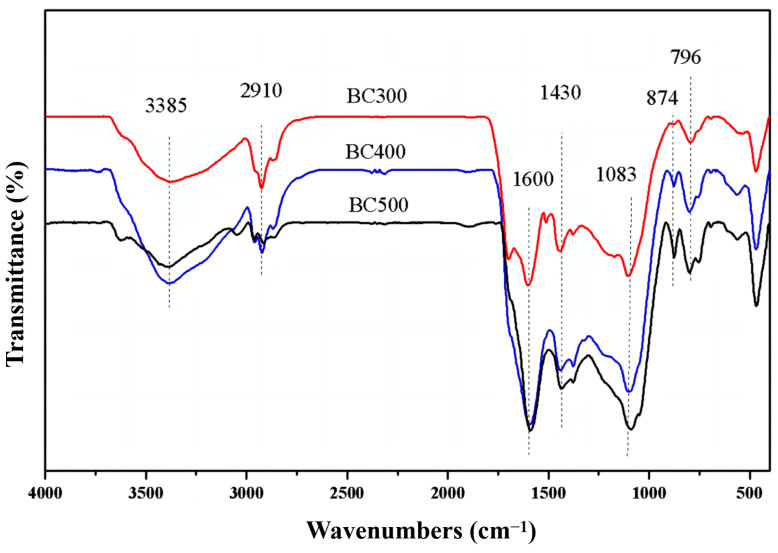
The near-infrared spectra of corn straw biochar at different temperatures.

Figure 1 presents a near-infrared spectral analysis of three distinct types of corn straw biochar within the wavenumber range of 4000–400 cm^−1^. The spectrum reveals that varying peak positions correspond to distinct functional groups, and the magnitude of the peaks indicates the relative abundance of these groups. A prominent and broad peak observed around 3385 cm^−1^ is attributed to the stretching vibration of hydroxyl -OH groups, which can be associated with either alcohols or acids [18]. Additionally, the antisymmetric stretching vibration of aliphatic CH_2_ groups is represented by the peak at 2910 cm^−1^ [19]. With an increase in the pyrolysis temperature, there is a noticeable trend of an initial increase followed by a subsequent decrease in the intensity of these two functional groups. Upon examination of Figure 1, it becomes evident that biochar produced at 400 °C (BC400) exhibits the highest content of these functional groups, surpassing that of BC300 and BC500. The presence of oxygenated functional groups is a critical determinant in the biochar’s capacity for heavy metal adsorption, as these groups facilitate interactions with metal ions, thereby enhancing the adsorptive properties of the biochar.

The aromatic ring skeleton C=C stretching vibration at 1590 cm^−1^ [18], the carbonate CO_3_^2−^ characteristic absorption peak at 1430 cm^−1^, the phosphate (PO_4_^3−^) characteristic absorption peak at 1083 cm^−1^ [20], the aromatic ring C-H deformation vibration absorption peaks at 874 and 796 cm^−1^ [18,21], and the C-C bond absorption peak at 463 cm^−1^ [19] are shown. The content of these functional groups was higher in BC500 than in BC300, indicating that the higher the preparation temperature, the greater the degree of aromatization of biochar [22,23], and at high temperatures, the inorganic mineral components were enriched, which is consistent with the variation pattern of biochar conductivity.

The order of absorbance of the characteristic absorption peak of phosphate (PO_4_^3−^) at 1083 cm^−1^ was BC400 > BC500 > BC300, which is different from the amount of detected effective phosphorus content because some phosphorus existed in the form of stable compounds at high temperatures, meaning BC500 has a higher amount of total phosphorus but a lower amount of available phosphorus. Cao et al. [24] prepared cow manure biochar at low temperatures (200 °C and 350 °C) and showed that although the total phosphorus content of the biochar was higher at 350 °C, the effective phosphorus content was lower, which may be related to the formation of stable P-Ca-Mg compounds at high temperatures.

Overall, the chemical structure and functional group composition of corn biochar changed with increasing treatment temperature, which affected its adsorption capacity for heavy metal ions. At lower temperatures, more hydroxyl functional groups may have provided better adsorption capacity, while at higher temperatures, although some functional groups may have decreased, structural reorganization may have provided new adsorption sites. The reasons for these differences may be related to temperature-induced chemical bond breaking, reorganization, and the thermal stability of the functional groups.

The specific surface area and total pore volume parameters of corn straw biochar at different temperatures are shown in Table 1. The specific surface area and pore volume in Table 1 were characterized by using nitrogen adsorption/desorption and BET methods (Surface Area and Porosity Analyzer, QuantachromeAutosorb iQ3M). It can be seen that the specific surface area and total pore volume decreased firstly and then increased with the increase in temperature, and the specific surface area was the largest in BC300 and the smallest in BC400, which might be due to the fact that the substances such as tar generated from the decomposition of organic matter damaged or blocked the pore structure of biochar during the temperature rise, leading to the decrease in its comparative area and total pore volume. When the temperature was increased to 500 °C, the pore structure of biochar became larger with the fuller combustion of organic matter, and thus, its specific surface area became larger.

Based on the above analysis, it can be found that BC400 had the best adsorption effect. The reasons can be summarized as follows: (1) BC400 had the highest EC value, and the content of water-soluble phosphorus was similar to the change mode of EC, indicating that water-soluble phosphorus may be one of the key sources of EC in biochar. The analysis also shows that BC400 had the highest content of water-soluble phosphorus. Phosphorus in the biochar reacts with heavy metals in the soil to form an insoluble sediment which then adsorbs heavy metals. (2) BC400 had the highest functional group content, while the oxygen-containing functional groups on the surface of biochar could combine with heavy metals to form a complex precipitation, which can adsorb heavy metals. (3) The order of absorbance of the characteristic absorption peak of phosphate (PO_4_^3−^) at 1083 cm^−1^ was BC400 > BC500 > BC300. Similar to the oxygen-containing functional groups, PO_4_^3−^ interacts with heavy metals in the soil to form a sediment and adsorb heavy metals.

### 2.2. Microstructure of Corn Straw Biochar Powder

**Figure 2:**
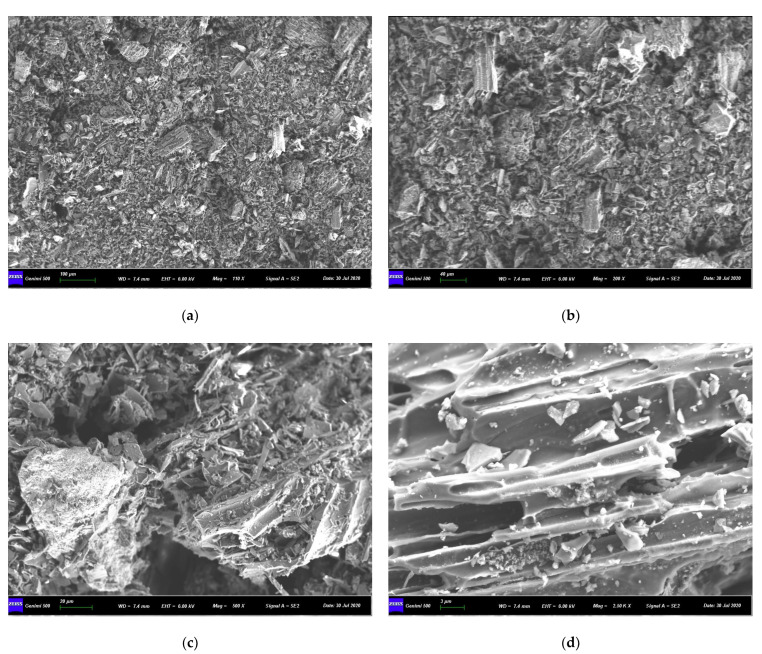

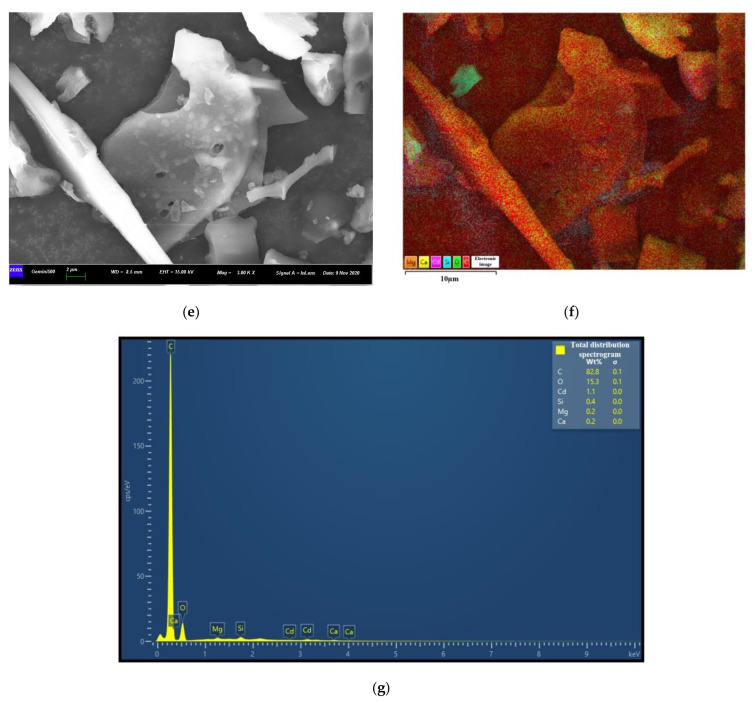
Micro-morphology and energy spectrum of corn straw biochar (BC400). (**a**) ×100; (**b**) ×40; (**c**) ×20; (**d**) ×3. (**e**–**g**) are the elemental distributions of biochar after adsorption of Cd^2+^.

A scanning electron microscope (SEM) was employed to observe the biochar powder (BC400) at the micron scale. As presented in Figure 2a–d, it can be seen from Figure 2a,b that under the condition of small magnification, the corn straw biochar is mainly composed of fine powder, which is mixed with irregular “fragments” as small as several microns. Combined with the analysis of biochar raw materials, it may be the fragments of corn straw calcined at high temperatures. It can also be seen from the figure that the structural arrangement of the corn straw biochar is not very close, which also provided a good basis for the adsorption of heavy metals by corn biochar. Compared with other people’s research [10,17,21], the shape of irregular “fragments” are significantly different, showing a block or strip shape. The reason for this phenomenon may be related to the roasting temperature or the different types of corn straw. By observing Figure 2c,d, it is found that there are many pore structures in the biochar, which provided a basis for the larger specific surface area of biochar. When observing the massive fragments at a higher magnification, it is found that there are some “flocculent” particles on the surface of the fragments, that is, “ash” particles rich in mineral components formed during the preparation of biochar, which are important points for adsorbing heavy metals. Minerals in corn straw biochar increase the content of internal cations, which will exchange with heavy metal ions in the soil solution and adsorb heavy metals. The morphology was observed and the energy spectrum at the typical locations was scanned to obtain the composition and distribution of the elements contained in the biochar. Figure 2e–g show the energy spectrum of some elements. It can be seen that in addition to organic elements such as C, H, and O, there are also components such as Ca, Mg, K, Si, and Fe in the corn straw biochar which could formally improve the soil’s pH value and conduct ion exchange adsorption, coprecipitation, and other important processes to remove heavy metals. The enrichment of Si and O in the corn char after adsorption of Cd was compared and corresponded to the distribution of Cd, indicating that the silica minerals contributed to the fixation of Cd.

### 2.3. Preparation and Analysis of Indoor Composite-Polluted Soil

The acidic soil samples tested were obtained from the 0–20 cm topsoil of farms near the ore-concentrated region of the Tongling mining area. The soil samples were naturally air-dried and sieved using a 20-mesh sieve after removing plant roots, stones, and other large impurities. The physicochemical properties of the test soil samples were determined, and the analytical results are presented in Table 2.

In order to avoid the error caused by the low concentration of heavy metals and to more accurately study the interaction between different heavy metals after the application of the soil remediation agent, in accordance with the risk control standard for agricultural land soil contamination (GB 15618-2018) [25], the test soil was treated with pollutant compounds comprising HgCl_2_, CdCl_2_, and Pb(NO_3_)_2_ solutions so that the content of Hg, Cd, and Pb in the polluted soil was 2.0, 1.5, and 400 mg/kg [26], respectively, and the soil water content was adjusted to 60–70% of the maximum water-holding capacity (WHC) in the field [27]. The samples were placed in an indoor ventilated environment for two weeks to allow for aging and culturing.

The total mass concentrations of Pb and Cd in the test soil were determined according to the standard GB/T 17140-1997 [28], and the concentrations of Pb and Cd in the digestion solution were determined using a WYS-2200 atomic absorption spectrophotometer. The total Hg content of the soil was directly measured by using a mercury measuring instrument (DMA-80 MILESTONE).

**Figure 3:**
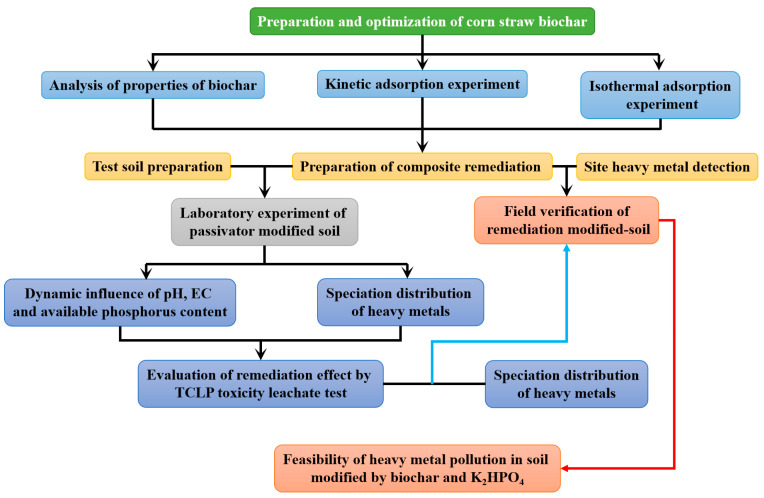
The experimental objectives and process of this study.

In order to clearly demonstrate the experimental objectives of this study, the experimental flow chart of this study is shown in Figure 3.

### 2.4. The Adsorption Potential of Biochar for Soil Heavy Metals

#### 2.4.1. Isothermal Adsorption Experiment

Pyrolysis temperature affects the physicochemical properties of biochar and its structural characteristics, thus affecting its absorbability. A recent study of Liu et al. [17,29] indicated that increasing the pyrolysis temperature (300–700 °C) improved the maximum adsorption capacity of silicon-rich rice husk biochar for soil Cd, mainly owing to more siliceous minerals promoting Cd precipitation. By conducting solution adsorption experiments (isothermal adsorption and kinetic adsorption), the adsorption potential and adsorption behavior of various biochars for Hg, Cd, and Pb were investigated [30], and the most suitable biochar pyrolysis temperature was determined.

In this study, stock solutions of 2000 mg/L Hg^2+^, Cd^2+^, and Pb^2+^ were prepared, the matrix was a 0.85 g/L NaNO_3_ solution, and the pH value was adjusted to 5.0 with dilute HNO_3_ and NaOH. All heavy metal solutions were obtained by diluting the corresponding stock solutions and using the same configuration scheme for the dilution solution.

**Figure 4:**
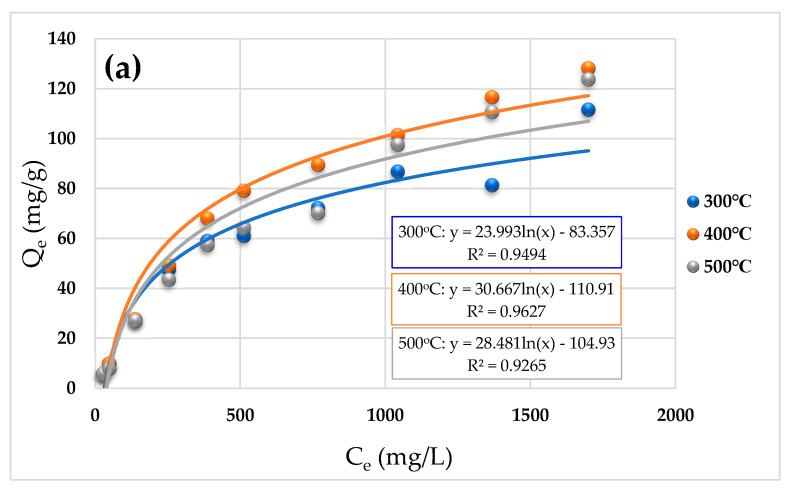

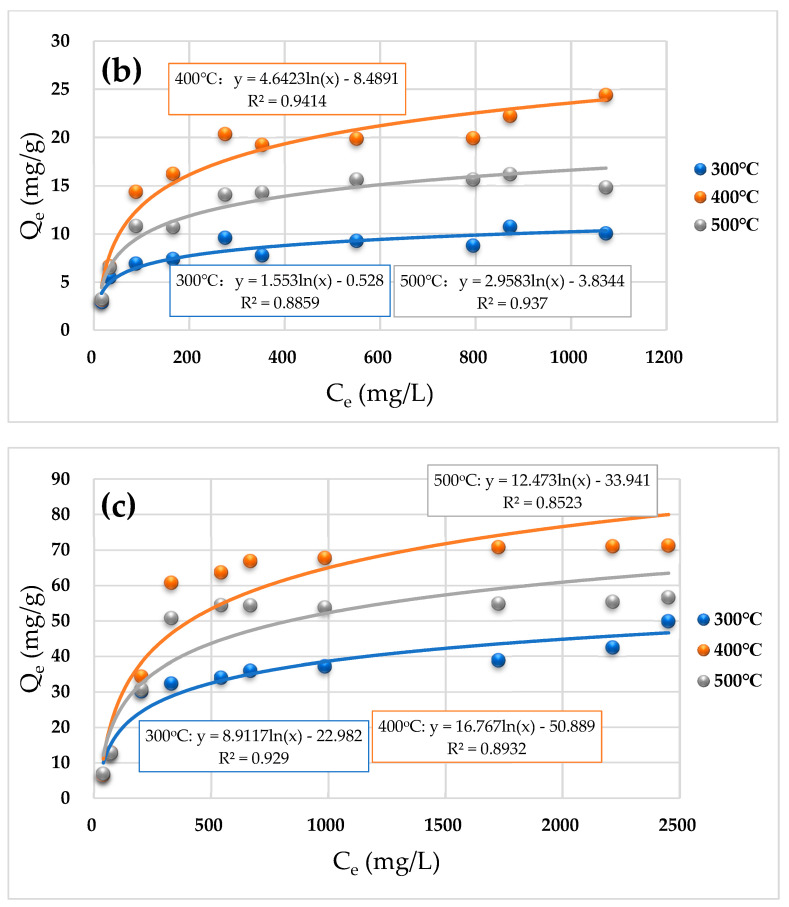
Adsorption isotherm curve of biochar for Hg^2+^ (**a**), Cd^2+^ (**b**), and Pb^2+^ (**c**).

The isotherm adsorption fitting curve is depicted in Figure 4, and the Langmuir fitting parameters of the isotherm adsorption experiment are listed in Table 3. The adsorption effects of biochar on Hg^2+^, Cd^2+^, and Pb^2+^ at different temperatures were similar, that is, BC400 > BC500 > BC300 (Figure 4), showing that they are connected to the physicochemical properties’ change laws of biochar such as conductivity and functional groups. Furthermore, the adsorption capacity of the three types of biochar for heavy metal ions increased progressively as the ion concentration in the solution increased. When the concentration reached a certain level, the adsorption capacity became steady. Cd^2+^ and Pb^2+^ displayed inflection points at 300 mg/L and 400 mg/L, respectively, but the curve changes of Hg^2+^ were inconspicuous. According to the composition of corn straw biochar, the reason for this may be that the soluble salts or oxygen-containing functional groups in the biochar used in this experiment were more tolerant to the heavy metal Hg^2+^ compared to the other two heavy metals, Cd^2+^ and Pb^2+^. Within a certain range, the ability to adsorb heavy metals was less affected with the increase in the concentration of heavy metal ions. It is speculated that if the concentration of the solution continues to increase, at the upper limit of Hg^2+^ concentration, there will be a state of saturation for the biochar, and then, an inflection point similar to the isothermal adsorption of Cd^2+^ and Pb^2+^ will appear.

In the Langmuir model, *Q_m_* represents the maximal adsorption capacity (Table 3). Except for Hg^2+^, where the adsorption capacity of BC500 was marginally higher than that of BC400, BC400’s adsorption capacity for the three heavy metal ions was higher. The better conductivity of BC400 may cause more soluble salts in the solution, intensifying the electrostatic attraction between metal ions and their negative surface charges. In addition, the content of functional groups and inorganic mineral components in BC400 was higher, promoting complexation and precipitation with metal ions, and the presence of functional groups enhanced the heavy metal adsorption sites. As a result, the mineral components improved the affinity of biochar for heavy metal ions.

#### 2.4.2. Kinetic Adsorption Experiment

**Figure 5:**
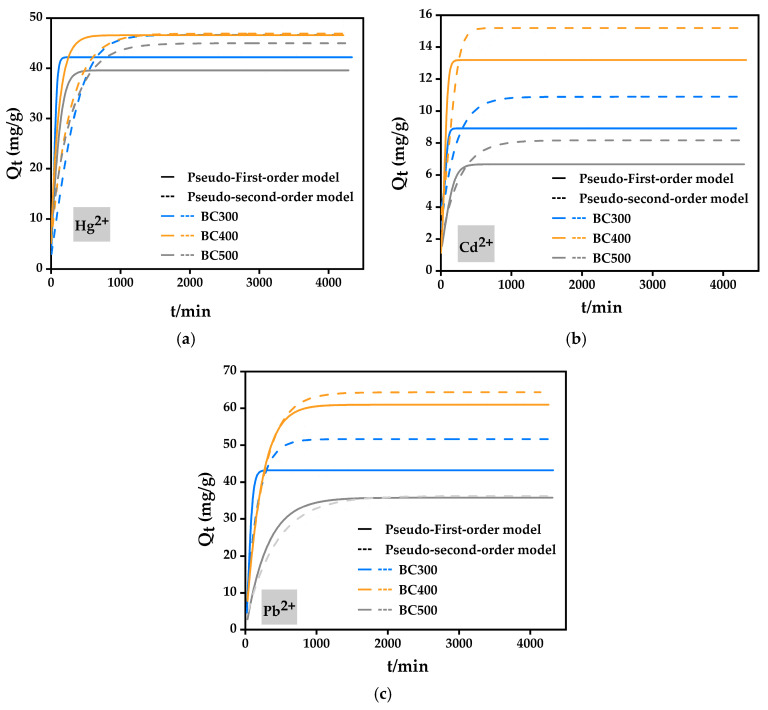
The adsorption kinetic curve of biochar for Hg^2+^ (**a**), Cd^2+^ (**b**), and Pb^2+^ (**c**).

Figure 5a–c are the fitting curves of the pseudo-first-order and pseudo-second-order kinetic models for the adsorption of Hg^2+^, Cd^2+^, and Pb^2+^ by three types of biochars (BC300, BC400, and BC500), respectively. After 24 h in the experiment, the adsorption of metal ions by biochars was almost balanced, and the equilibrium adsorption capacity regulations of the three types of biochars were basically consistent with the isothermal adsorption results, that is, BC400 > BC500 > BC300. Moreover, the pseudo-two-order kinetic model was obviously superior to the pseudo-first-order kinetic model. The corresponding fitting parameters of the two dynamic equations are depicted in Table 4.

Table 4 shows that the R^2^ value of the pseudo-first-order kinetic model was much higher than that of the pseudo-second-order kinetic model on the whole, which indicates that the adsorption of metal ions Hg^2+^, Cd^2+^, and Pb^2+^ by corn straw biochar was mostly a chemical reaction process. The actual equilibrium adsorption amounts of metal ions Hg^2+^, Cd^2+^, and Pb^2+^ corresponding to BC300, BC400, and BC500 were 49.49, 49.43, and 47.00 mg/g, 8.60, 15.70, and 11.28 mg/g, and 38.18, 66.72, and 55.88 mg/g, respectively. Compared with the *q_e_* fitted by the kinetic model, the result fitted by the pseudo-second-order equation was closer to the actual adsorption capacity, further demonstrating that the pseudo-second-order model was more suitable for elucidating the adsorption process of metal ions by biochar. The same conclusion can be obtained by comparing the research results of others on the adsorption of copper by corn straw biochar [13].

BC400 showed the best adsorption effect on Hg^2+^, Cd^2+^, and Pb^2+^ in the isothermal and kinetic adsorption studies. As a result, BC400 was selected as the optimal biochar for subsequent soil remediation experiments.

### 2.5. Near-Infrared Spectra of Biochar before and after Adsorption of Metal Ions

**Figure 6:**
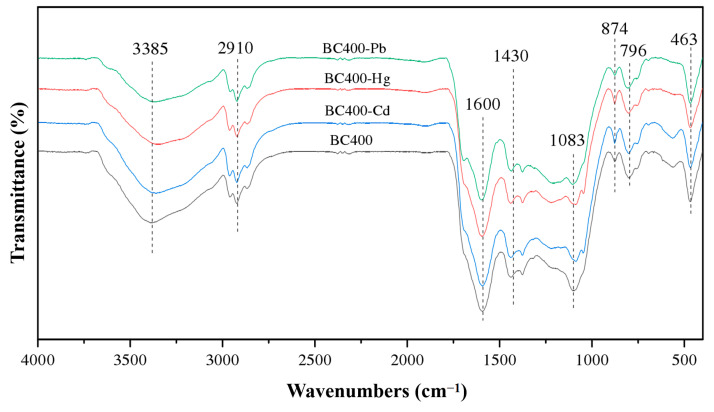
Near-infrared spectra of BC400 before and after adsorption of heavy metals.

As shown in Figure 6, after the adsorption of Hg^2+^ and Pb^2+^ by BC400, the transmittance of the hydroxyl -OH absorption peak at 3385 cm^−1^ was decreased by about 0.08 and 0.13, respectively, indicating that -OH on the surface of the adsorbent was involved in the adsorption reaction. The transmittance of the absorption peak at 2910 cm^−1^ decreased by 0.02 and 0.04, respectively, suggesting that -COOH also reacted with Hg^2+^ and Pb^2+^ [6,17]. The parallel change in the transmittance of the C=C absorption peak at 1613 cm^−1^ indicated that Hg^2+^ and Pb^2+^ ions could undertake surface complexation with the delocalized π electron of BC400. The CO_3_^2−^ absorption peak at 1430 cm^−1^ and the PO_4_^3−^ absorption peak at 1083 cm^−1^ were slightly shifted and the transmittance was decreased, indicating that Hg^2+^ and Pb^2+^ might combine with CO_3_^2−^ and PO_4_^3−^ to generate an insoluble precipitate [19].

By comparing the spectra of the original biochar and the adsorbed Cd^2+^ ions in Figure 6, it can be seen that the absorption peaks of multiple functional groups in the spectra before and after the adsorption of Cd^2+^ by BC400 were basically unchanged, indicating that the various functional groups in the corn straw biochar used in this study had no contribution to the adsorption and fixation of Cd^2+^, which is also the reason for the insignificant adsorption of Cd^2+^ ions.

### 2.6. Laboratory Experiment of Biochar- and KH_2_PO_4_-Passivated Soil

#### 2.6.1. Dynamic Effect of Soil’s Physicochemical Properties

(1) Dynamic effect of pH value

**Figure 7:**
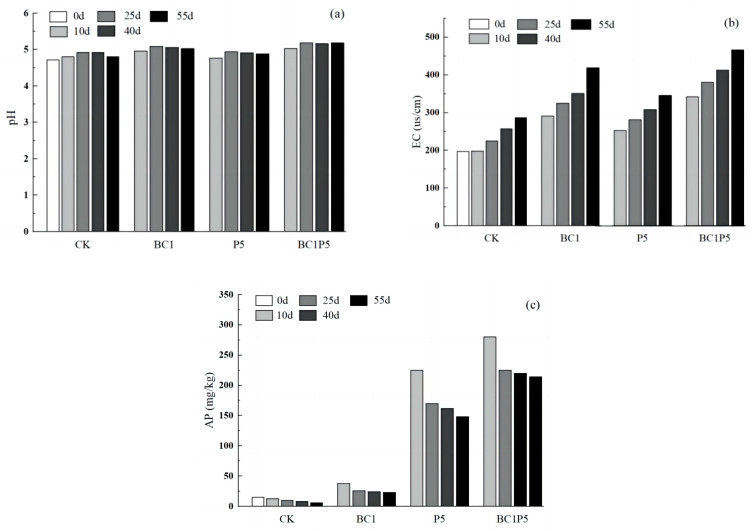
Changes in pH (**a**), EC (**b**), and available phosphorus (**c**) of soils treated with different remediation agents.

The changes in soil pH following various remediation treatments are shown in Figure 7a. The original pH value of the contaminated soil sample was 4.72, and the pH values’ fluctuation range of the CK group during the culture period was about 0.2. The soil pH values of the BC1, P5, and BC1P5 treatment groups on the 55th day of culture were 5.03, 4.88, and 5.18, respectively. Compared to the CK group, the soil pH values of the biochar-added treatments all increased to varying degrees, and the P5 treatment soil’s pH values were similar to those of the CK group. The release of K, Na, Ca, Mg, and other basic cations in the biochar and its contained alkaline functional groups increased soil pH, increasing the stability of heavy metals in the soil [31,32]. Weng et al. [33] discovered that alkaline biochar and red mud could increase soil pH and the negative charge on the soil surface and enhance the heavy metal cations’ precipitation on the soil surface. The addition of KH_2_PO_4_ had little effect on soil pH, probably because the introduced H^+^ was within the action range of the soil’s acid–base buffer system [34].

(2) Dynamic effect of EC

The EC reflects the strength of soil ions [35], and the dynamic changes in EC in different treatment groups are depicted in Figure 7b. The experimental results revealed that the addition of biochar significantly increased the EC of contaminated soil. During the culture period, the EC of the CK, BC1, P5, and BC1P5 treatment groups was 197~287, 291~419, 253~346, and 342~466 μs/cm, respectively. The EC of all treatment groups, including the CK group, increased with increased culture time. The soil EC was increased in all three passivator treatment groups, and the order of increasing amount was BC1P5 > BC1 > P5.

(3) Dynamic effect of available phosphorus content

The changes in soil available phosphorus content during the cultivation period are shown in Figure 7c. The available phosphorus content of all treatment groups decreased with increasing culture time, which may be due to the phosphate brought in by the soil itself or to the addition of the curing agent, which may react with heavy metal elements through precipitation, reducing the available phosphorus content in the contaminated soil [17,36]. On the 55th day of culture, the available phosphorus content in the BC1, P5, and BC1P5 treatment groups increased by 17.34, 140.82, and 199.49 mg/kg, respectively, compared to the CK group. This demonstrates that both biochar and KH_2_PO_4_ can increase the content of available phosphorus in the soil, and the combined application of the two is better than the sum of the two applications alone. This could be because too much phosphate was introduced into the soil when the two treatments were applied simultaneously, and it could not be fully utilized in such a short time, so it remained in the soil as available phosphorus and gradually reacted with heavy metal ions in the soil as culture time increased [37].

Overall, biochar and KH_2_PO_4_ increased soil pH, EC, and available phosphorus content, decreased the competition between H^+^ and Al^3+^ in the soil for biochar adsorption sites, and enhanced the ion exchange through the precipitation reaction, therefore boosting the adsorption of metal elements.

#### 2.6.2. Effect on the Distribution of Heavy Metals in Soil

After adding biochar to the soil, biochar itself may contain a certain amount of heavy metals such as Hg, Cd, or Pb, which will cause errors when mixed with the actual results. According to the results of the energy spectrum analysis of biochar in Section 2.3, in addition to organic elements such as C, H, and O, the corn straw biochar also contains elements such as Ca, Mg, K, Si, and Fe, and does not contain heavy metals such as Hg, Cd, and Pb, which will not affect the subsequent experimental results.

**Figure 8:**
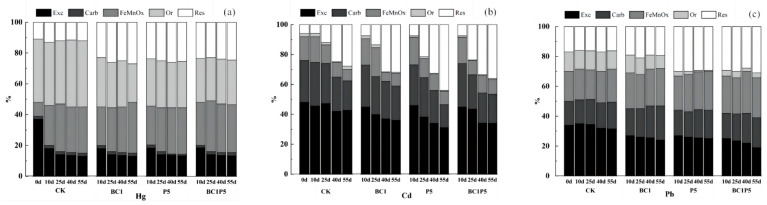
Changes in soil heavy metal speciation after different treatments: Hg (**a**), Cd (**b**), and Pb (**c**). Exc-: exchangeable state, Carb-: carbonate-bound state, FeMnO*_x_*-: iron and manganese oxide-bound state, Or-: organic state, and Res-: residue state.

The bioavailability and mobility of metal elements are reflected in the content of various forms of heavy metals in contaminated soil [38]. The morphological changes in Hg, Cd, and Pb in the soil over the cultivation period are shown in Figure 8. With the addition of the passivating agent, the heavy metals in the soil as a whole shifted from a relatively active form to a more stable form as the culture period was extended.

The primary forms of Hg in the soil were FeMnO*_x_*-Hg and Or-Hg from the CK group, as illustrated in Figure 8a. The soil’s fixation mainly promoted the conversion of Exc-Hg into FeMnO*_x_*-Hg, and the Hg forms were essentially stable after 40 days of culture. Compared to the CK group, the three treatments groups of BC1, P5, and BC1P5 shifted Or-Hg to Res-Hg. On the 55th day, Or-Hg fell by 16%, 11%, and 11%, respectively, whereas Res-Hg grew by 14%, 13%, and 11%, indicating that biochar application alone had the best curing effect on Hg. The addition of biochar or KH_2_PO_4_ could reduce the proportion of Exc-Hg in soil and inhibit the conversion of Or-Hg into its active form, hence increasing the content of Res-Hg and gradually converting active Hg to inert Hg.

As depicted in Figure 8b, the primary forms of Cd in the soil from the CK group were Exc-Cd and Carb-Cd, with the highest content of Exc-Cd being 48–43%, and the lowest content of Or-Cd being 1–2%, and the solidification ability of the soil itself converted Exc-Cd, Carb-Cd, and FeMnO*_x_*-Cd to Res-Cd. Compared to the CK group, the three groups of BC1, P5, and BC1P5 transformed Exc-Cd to Res-Cd, with Exc-Cd decreasing by 7%, 15%, and 10%, respectively, and Res-Cd increasing by 4%, 16%, and 9% on the 55th day of culture, indicating that KH_2_PO_4_ added alone had the best effect on Cd curing. Phosphorus and Cd have a strong association in soil, and phosphorus-containing substances can affect the availability of soil Cd through a series of mechanisms.

According to the CK group, the primary form of Pb in the soil was Exc-Pb (34–31%), and the percentage variations of each form during the culture period were minor (Figure 8c). Compared to the CK group, the three treatments of BC1, P5, and BC1P5 transformed Exc-Pb and Or-Pb into Carb-Pb, FeMnO*_x_*-Pb, and Res-Pb, and on the 55th day of culture, Exc-Cd decreased by 9%, 6%, and 15%, respectively, Carb-Pb increased by 6%, 1%, and 3%, FeMnO*_x_*-Pb increased by 3%, 4%, and 6%, Or-Pb decreased by 4%, 11%, and 11%, respectively, and Res-Pb increased by 3%, 14%, and 17%, indicating that the combined application of Pb-fixed biochar and KH_2_PO_4_ had the best effect. Cao et al. [39] validated through in situ repair studies that phosphate could significantly lower the content of available Pb required to convert Pb from a carbonate-bound state to a residual state, with residual Pb increasing by up to 53%. Adding phosphate to contaminated soil can drastically reduce the effective concentration of heavy metals and promote the transformation of heavy metals to tightly bound states [40], with Pb being the most prominent example.

To summarize, the fixation effect of the composite passivator occurred when the two agents were applied separately for Hg and Cd adsorption. For Pb adsorption, the composite passivator had a more significant fixing impact than the sum of the two applied separately, consistent with the effect on soil available phosphorus content, reflecting that phosphate precipitation phenomena had a more noticeable effect on Pb immobilization. The competitive adsorption of several heavy metals in composite-contaminated soil may increase the activity of particular heavy metals [41]. Pb has a higher atomic weight and greater electronegativity than Hg and Cd elements, promoting Pb remediation while preventing biochar’s Hg and Cd adsorption and precipitation [17,42].

Compared to a single application, the synergistic effect of the combined treatment of biochar and KH_2_PO_4_ on reducing heavy metal Exc- and increasing Res- was discernible. However, on the premise of ensuring the remediation repair effect, the combined treatment could compensate for soil acidification caused by acid phosphate, thus having more application advantages.

### 2.7. Evaluation of the Remediation Effect of Biochar- and KH_2_PO_4_-Passivated Soil

The corresponding heavy metal contents in the TCLP leachate are listed in Table 5. When biochar was applied alone, the Cd and Pb contents in the BC1 treatment group’s leachate were reduced by 6.65% and 10.99%, respectively, compared to the CK group. When KH_2_PO_4_ was applied alone, the Cd and Pb contents in the leachate of the P5 treatment group were reduced by 4.22% and 22.77% compared to the CK group. When biochar and KH_2_PO_4_ were applied simultaneously, the contents of Cd and Pb in the BC1P5 treatment group’s leachate were reduced by 10.90% and 32.46%, respectively, compared to the CK group. When the two curing agents were applied individually, the reductions were close to the total of the corresponding decreases. The magnitude of Hg content in each leachate remained essentially constant.

In the composite-contaminated soil, the content of heavy metals in the leachate of each group was lower than the corresponding standard threshold. Compared to the CK group, the contents of Cd and Pb in the leachate of each group in the composite-contaminated soil dropped by varying degrees, with Pb content decreasing more remarkably than Cd concentration. The toxic leaching level of Hg was unaffected by soil pollution types and treatment methods, and it was lower than the standard’s mercury limit of 0.10 mg/L.

### 2.8. Field Demonstration Study of Biochar- and KH_2_PO_4_-Passivated Soil

**Figure 9:**
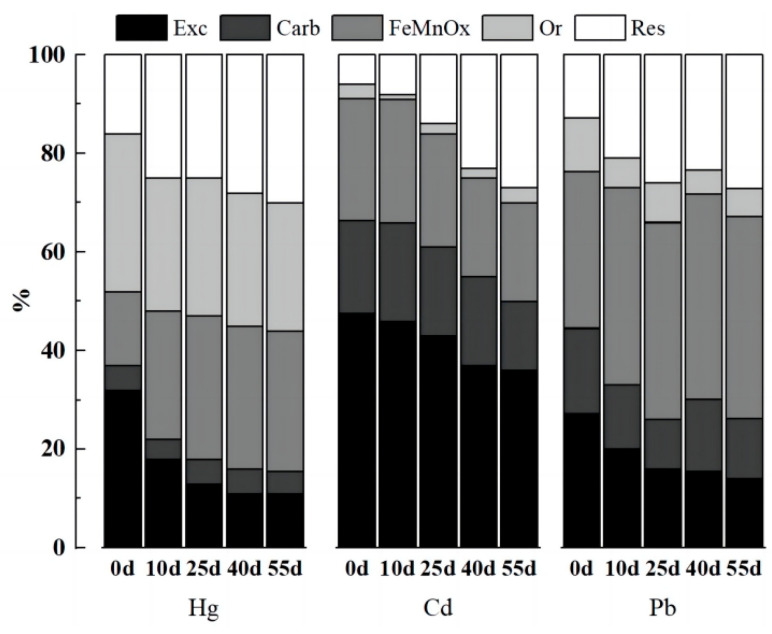
Morphological changes of heavy metals Hg, Cd, and Pb in soil after different treatments.

The morphological changes of Hg, Cd, and Pb in the soil over the experimental period are shown in Figure 9. The passivator mainly converted Exc-Hg, the current form of Hg in the soil, into FeMnO*_x_*-Hg. After adding the passivator for 55 days, Res-Hg increased by 14.24%, and Exc-Hg decreased by 20.12%. Exc-Cd, the current Cd form, was converted into a stable state (Res-Cd). After adding a passivator for 55 days, Res-Cd increased by 20.25%, Exc-Cd decreased by 11.82%, Res-Pb (the current form of Pb) climbed by 14%, and Exc-Pb declined by 15.31%. With the prolongation of culture time, Hg, Cd, and Pb all showed an increase in the proportion of stable states and a decrease in exchangeable states, and the bioavailability of heavy metals decreased significantly. This shows that the combined passivator of biochar and KH_2_PO_4_ also has an excellent passivation effect on outdoor heavy metal-contaminated soil.

## 3. Materials and Methods

### 3.1. Preparation of Composite Materials

In this study, biochar and KH_2_PO_4_ were selected as composite materials, and corn straw biochar (ITIGCN Corp, China; patent approval number: 200920232191.9) was prepared through pyrolysis at three temperatures of 300, 400, and 500 °C and recorded as BC300, BC400, and BC500. The pyrolysis process was carried out under a nitrogen atmosphere and limited oxygen conditions; nitrogen was used to ensure an oxygen-free environment. And the holding time of each biochar sample at the specified temperature was 2 h. The KH_2_PO_4_ used in this study was of GR (guaranteed reagent) grade. The pH and electrical conductivity (EC) of biochar were determined using a G23 multi-parameter analyzer, and the carbon-to-water ratio was 1:10 [16]. The ash content was determined using the burning technique, which was based on the test method of wood charcoal (GB/T17664-1999) [43]. The phosphorus content in the aqueous biochar solution was determined using the molybdate–ascorbic acid colorimetric technique, and the carbon-to-water ratio was 1:10. The surface functional group characterization of biochar was determined using a VERTEX 70 BRUKER Fourier transform infrared spectrometer.

### 3.2. Preparation of Indoor Composite-Polluted Soil

The pH and EC of the soil were measured using a G23 multi-parameter tester with a solid–liquid ratio of 1:5 and 1:10, respectively. The maximum field water-holding capacity was determined by using the ring knife method for the standard NY/T 1121.22-2010 [44]. As the test soil was acidic, the available phosphorus content was determined according to the industry-standard NY/T 1121.7-2006 [45] method, with a solid–liquid ratio of 1:10. The organic matter content was determined according to the standard NY/T 1121.6-2006 [46].

### 3.3. Isothermal Adsorption Experiment

Hg^2+^, Cd^2+^, and Pb^2+^ were diluted with the matrix to make 0.15, 0.30, 0.75, 1.50, 2.50, 3.00, 4.50, 6.00, 7.50, and 9.00 mol/L Hg^2+^, Cd^2+^, and Pb^2+^ solutions, respectively; we accurately weighed 0.125 g of BC300, BC400, and BC500 into a 50 mL centrifuge tube, and 25 mL of Hg^2+^, Cd^2+^, and Pb^2+^ solutions with varied contents was added, respectively, mixed well, and shaken in a constant-temperature water bath at 25 °C for 24 h. The heavy metal ion contents of the solutions were measured after centrifugal filtration and dilution.

The isotherm adsorption model can reflect the adsorption properties of the adsorbent and its mechanism on heavy metal ions. The Langmuir model is more appropriate for fitting biochar isotherm adsorption data to Hg^2+^, Cd^2+^, and Pb^2+^. In this study, the Langmuir model was used for fitting. The model formula is

$$q_{e}=q_{max}*\frac{K_{L}C_{e}}{1+K_{L}C_{e}}$$

where *q_e_* is the adsorption capacity (or loading) and the quantity of adsorbate taken up by the adsorbent per unit mass (or volume) at adsorption equilibrium (mg/g). *q_max_* is the maximum adsorption capacity at adsorption equilibrium (mg/g), related to the adsorption site. *K_L_* is the Langmuir’s equilibrium adsorption constant (mg/L); *C_e_* is the equilibrium concentration of the contaminants in the solution.

### 3.4. Kinetic Adsorption Experiment

In this study, 1.5 mol/L Hg^2+^, Cd^2+^, and Pb^2+^ solutions were prepared by diluting the matrix. A total of 0.125 g of BC300, BC400, and BC500 was accurately weighed into a 50 mL centrifuge tube, and then 25 mL of Hg^2+^, Cd^2+^, and Pb^2+^ solutions was shaken at 25 °C for 0, 5, 10, 20, and 30 min at 1, 2, 4, 6, 12, 24, 48, and 72 h after sampling to determine the contents of Hg^2+^, Cd^2+^, and Pb^2+^ in the solution.

The study of adsorption kinetics elucidates the process of solute mass transfer at the interface between solid and liquid phases, offering insights into the mechanisms behind multiphase reactions and chemical adsorption. To comprehend the rate at which biochar adsorbs metal ions and to manage the reaction’s equilibrium time, two models were employed: the pseudo-first-order kinetics model and the pseudo-second-order kinetics model. These models were used to fit the experimental data of adsorption kinetics, and the fitting efficacy of each model was assessed by comparing the experimental data with the calculated data and examining the regression coefficient (*R*^2^).

The pseudo-first-order and pseudo-second-order kinetic equations are as follows:

$$q_{t}=q_{e}(1-e^{{-k}_{1}t})$$

$$q_{t}={q_{e}}^{2}k_{2}t/(1+q_{e}k_{2})$$

where *q_t_* is the adsorption capacity (mg/g) at *t*. *q_e_* is the equilibrium adsorption capacity (mg/g). *k*_1_ is the rate constant of first-order kinetics (g (mg·min)^−1^). *k*_2_ is the rate constant of second-order kinetics (g (mg·min)^−1^).

### 3.5. Near-Infrared Spectra of Biochar before and after Adsorption of Metal Ions

The dried biochar sample was prepared in the form of KBr tablets, and then the standard substance (such as pure KBr) was used to calibrate the infrared spectrometer to ensure that the instrument was in the best working condition; the background was scanned without the sample, and the background spectrum was recorded so that the background signal could be deducted from the sample spectrum later. Finally, the dried biochar sample powder was mixed with KBr powder and made into transparent flakes under high pressure using a tablet press. The sample was placed in the light path of an infrared spectrometer, and the infrared spectrum of the sample was recorded. In this study, a Nicolet 8700 Fourier Transform Infrared Spectrometer was used to detect the functional groups of the biochar. The main parameters of the instrument include a spectral range of 7500-50 cm^−1^, a resolution of 0.1 cm^−1^, and a wavenumber accuracy of 0.01 cm^−1^.

The changes in the corresponding characteristic peak positions of each group in the spectra before and after adsorption were analyzed by comparing the near-infrared spectra of BC400 before and after adsorption of heavy metals Hg^2+^, Cd^2+^, and Pb^2+^; this would elucidate the correlation between metal ion adsorption and the functional groups contained in the adsorbent, and would enable us to generalize the possible adsorption mechanism.

### 3.6. Laboratory Experiment of Biochar- and KH_2_PO_4_-Passivated Soil

In the experiment, 500 g of contaminated soil samples was weighed into a plastic container. The soil that was not treated with a curing agent served as the control (CK). Three treatments were established for the remediation experiment: BC1 had 1% biochar added (as a percentage of the mass ratio of biochar to dry soil), P5 had 5 molar ratios of KH_2_PO_4_ added (based on the ratio of the molar mass of phosphorus to heavy metals), and BC1P5 had both 1% biochar and 5 molar ratios of KH_2_PO_4_ added. After the passivating agents were added, the mixture was thoroughly blended, maintaining 60–70% of the maximum water-holding capacity. Samples were extracted after 10, 25, 40, and 55 days of incubation. The samples were then naturally air-dried, ground, sieved, and tested for various physicochemical properties of the soil (pH, electrical conductivity, and available phosphorus) and the content of heavy metals in different chemical forms.

The chemical speciation analysis of heavy metals adopted an improved Tessier multistage continuous extraction method, and the specific extraction steps are stated in [47]. The morphology of each level was divided into 5 categories: Exc-, Carb-, FeMnOx-, Or-, and Res-. Atomic absorption spectrometry was used to determine the contents of Pb and Cd in the morphologically fractionated extract, and a PF5 atomic fluorescence spectrometer was used to determine the content of Hg.

### 3.7. Evaluation of the Remediation Effect of Biochar- and KH_2_PO_4_-Passivated Soil

To evaluate the fixation effect of biochar and KH_2_PO4 on Hg, Cd, and Pb, the soil samples after 55 days of culture were subjected to a toxicity characteristic leaching procedure (TCLP) treatment to identify the leaching features of heavy metal elements in the soil after the passivator curing treatment. When the soil samples were leached by using the TCLP, an acetic acid buffer aqueous solution with a pH of 4.9 was utilized as the leaching solution.

### 3.8. Field Demonstration Study of Biochar- and KH_2_PO_4_-Passivated Soil

Similar to the above biochar adsorption experiment, HgCl_2_, CdCl_2,_ and Pb (NO_3_)_2_ solutions were used to conduct combined pollution treatments on the test soil so that the concentrations of Hg, Cd, and Pb in the soil were 0.59, 0.96, and 176 mg/kg, respectively, making the experimental site reflect a relatively heavy metal pollution state. Then, we started the next step.

Large chunks of soil were pulverized and sprayed with a passivating chemical at the experimental site. The passivating ingredient was combined with 1% BC400 biochar and a 5-molar ratio KH_2_PO_4_ mixture. After mixing the passivating agent with the contaminated soil at a 0–20 cm depth, the field was leveled. After adding the passivator, samples were obtained after 0 d, 10 d, 25 d, 40 d, and 55 d. The sampling spots were determined using the plum blossom method, and samples of 0–20 cm topsoil were taken. Finally, the soil samples from different areas were mixed into a single soil sample, and then the soil samples were broken up and thoroughly mixed after removing stones, plant roots, and other impurities. The soil samples were naturally air-dried and sieved using a 20-mesh sieve before being analyzed for the presence of various chemical forms of heavy metals such as Hg, Cd, and Pb.

## 4. Conclusions

In this study, indoor and outdoor experiments were conducted to investigate the effects of biochar and potassium dihydrogen phosphate (KH_2_PO_4_) on the immobilization and remediation of heavy metals such as Hg^2+^, Cd^2+^, and Pb^2+^ in soil. The results indicated that BC400 exhibited the most effective adsorption capacity in isothermal and kinetic adsorption studies, and thus, it was selected as the optimal biochar material for subsequent soil remediation experiments. In addition, this study underscores the significance of developing straightforward, cost-effective, and efficient soil remediation techniques. It highlights that chemical remediation methods represent a viable approach to addressing heavy metal-contaminated soil. However, it is crucial to acknowledge the limitations of various methods and to consider the potential benefits of combining various remediation agents to enhance the efficacy of soil remediation. Although there are relatively few studies on the use of biochar and phosphate fertilizer composites for soil remediation, this mixed-additive remediation technology has the potential to be applied for the remediation of complex and changeable heavy metal pollutants in natural soil. Future research should further explore the adsorption mechanism under different conditions, optimize the proportion and application amount of combined additives, and evaluate the long-term stability and environmental impact in order to promote the practical application and development of this technology.

## Figures and Tables

**Table 1:** Basic physical and chemical properties of corn straw biochar.

Biochar	pH	Ash * (%)	EC (ms/cm)	Water-Soluble Phosphorus (mg/kg)	Specific Surface Area (m^2^/g)	Total Pore Volume (cm^3^/g)
BC300	7.49 ± 0.02	21.31 ± 2	3.22 ± 0.5	477.33 ± 95	29.6	0.03295
BC400	8.95 ± 0.02	22.78 ± 2	5.74 ± 0.5	596.75 ± 95	12.1	0.02517
BC500	9.85 ± 0.02	24.54 ± 2	2.79 ± 0.5	233.53 ± 95	23.3	0.03170

*: Dry ash-free basis; EC, electrical conductivity.

**Table 2:** Physical and chemical properties of soil tested.

pH	EC * (μs/cm)	WHC * (%)	AP * (mg/kg)	OM * (g/kg)	Total Hg (mg/kg)	Total Cd (mg/kg)	Total Pb (mg/kg)
4.81	97	29.51	14.80	28.87	0.15	0.28	76.94

*: EC, electrical conductivity; WHC, water-holding capacity; AP, available phosphorous; OM, organic matter.

**Table 3:** Langmuir fitting parameters and their standard deviation of adsorption of Hg^2+^, Cd^2+^, and Pb^2+^ by biochar.

Metal Ions	Specimen Types	*Q_m_* * (mg/g)	*K_L_* (L/mg)	*R* ^2^
Hg^2+^	BC300	129.31 ± 25.00	0.00192 ± 0.0004	0.963
	BC400	173.27 ± 25.00	0.00152 ± 0.0004	0.995
	BC500	190.24 ± 25.00	0.00100 ± 0.0004	0.985
Cd^2+^	BC300	9.97 ± 5.00	0.02700 ± 0.009	0.964
	BC400	22.83 ± 5.00	0.00656 ± 0.009	0.965
	BC500	17.59 ± 5.00	0.02213 ± 0.009	0.975
Pb^2+^	BC300	45.45 ± 15.00	0.00457 ± 0.002	0.938
	BC400	81.47 ± 15.00	0.00479 ± 0.002	0.942
	BC500	56.84 ± 15.00	0.00010 ± 0.002	0.979

*: *Q_m_*, maximal adsorption capacity.

**Table 4:** Fitting parameters and their standard deviation of the pseudo-first-order model and the pseudo-second-order model for the adsorption of Hg^2+^, Cd^2+^, and Pb^2+^ by biochar.

Metal Ions	Specimen Types	Pseudo-First-Order Dynamic Model			Pseudo-Second-Order Dynamic Model		
		*Q_e_* (mg/g)	*K*_1_ (min^−1^)	*R* ^2^	*Q_e_* (mg/g)	*K*_2_ (mg(g∙min)^−1^)	*R* ^2^
Hg^2+^	BC300	37.58 ± 3.50	0.0025 ± 0.007	0.7553	33.52 ± 1.00	0.0001 ± 0.00005	0.6801
	BC400	46.18 ± 3.50	0.0215 ± 0.007	0.7392	31.73 ± 1.00	0.0002 ± 0.00005	0.6539
	BC500	43.12 ± 3.50	0.0108 ± 0.007	0.8020	35.10 ± 1.00	0.0001 ± 0.00005	0.6785
Cd^2+^	BC300	8.20 ± 5.00	0.0118 ± 0.01	0.5367	5.33 ± 3.00	0.0005 ± 0.00005	0.7175
	BC400	20.86 ± 5.00	0.0276 ± 0.01	0.7386	12.58 ± 3.00	0.0005 ± 0.00005	0.6657
	BC500	14.82 ± 5.00	0.0443 ± 0.01	0.6995	8.79 ± 3.00	0.0006 ± 0.00005	0.6812
Pb^2+^	BC300	25.18 ± 7.00	0.0025 ± 0.015	0.7217	22.63 ± 9.00	0.0001 ± 0.00004	0.6302
	BC400	51.17 ± 7.00	0.0422 ± 0.015	0.5751	44.10 ± 9.00	0.0002 ± 0.00004	0.6850
	BC500	73.24 ± 7.00	0.0406 ± 0.015	0.7179	35.89 ± 9.00	0.0001 ± 0.00004	0.6763

**Table 5:** Concentrations and standard deviation of heavy metals in toxicity leachate of composite-contaminated soil.

Specimen Types	Hg (mg/L)	Cd (mg/L)	Pb (mg/L)
CK	0.0104 ± 0.00008	0.0131 ± 0.0005	4.5503 ± 0.600
BC1	0.0102 ± 0.00008	0.0122 ± 0.0005	4.0503 ± 0.600
P5	0.0103 ± 0.00008	0.0125 ± 0.0005	3.5144 ± 0.600
BC1P5	0.0102 ± 0.00008	0.0117 ± 0.0005	3.0733 ± 0.600

## Data Availability

The data are contained within the article.
